# Screening of Altered Metabolites and Metabolic Pathways in Celiac Disease Using NMR Spectroscopy

**DOI:** 10.1155/2021/1798783

**Published:** 2021-11-15

**Authors:** Ensieh Khalkhal, Mostafa Rezaei-Tavirani, Fariba Fathi, B. Fatemeh Nobakht M. Gh, Amir Taherkhani, Mohammad Rostami-Nejad, Nastaran Asri, Mohammad Hossain Haidari

**Affiliations:** ^1^Proteomics Research Center, Faculty of Paramedical Sciences, Shahid Beheshti University of Medical Sciences, Tehran, Iran; ^2^Biochemistry Department, University of Wisconsin-Madison, Madison, WI 53706, USA; ^3^Chemical Injuries Research Center, Systems Biology and Poisoning Institute, Baqiyatallah University of Medical Sciences, Tehran, Iran; ^4^Research Center for Molecular Medicine, Hamadan University of Medical Sciences, Hamadan, Iran; ^5^Gastroenterology and Liver Diseases Research Center, Research Institute for Gastroenterology and Liver Diseases, Shahid Beheshti University of Medical Sciences, Tehran, Iran

## Abstract

**Background:**

Celiac disease (CeD) is an autoimmune intestinal disorder caused by gluten protein consumption in genetically predisposed individuals. As biopsy sampling is an invasive procedure, finding novel noninvasive serological markers for screening of at-risk CeD population is a priority. Metabolomics is helpful in monitoring metabolite changes in body fluids and tissues. In the present study, we evaluated serum metabolite levels of CeD patients relative to healthy controls with the aim of introducing new biomarkers for population screening.

**Method:**

We compared the serum metabolic profile of CeD patients (*n* = 42) and healthy controls (*n* = 22) using NMR spectroscopy and multivariate analysis.

**Result:**

25 metabolites were identified by serum metabolic profiling. Levels of 3-hydroxyisobutyric acid and isobutyrate showed significant differences in CeD patients' samples compared with healthy controls (*p* < 0.05). According to pathway analysis, our data demonstrated that changes in nine metabolic pathways were significantly disrupted/affected in patients with CeD. These enriched pathways are involved in aminoacyl-tRNA biosynthesis; primary bile acid biosynthesis; nitrogen metabolism; glutamine and glutamate metabolism; valine, leucine, and isoleucine biosynthesis and degradation; taurine and hypotaurine metabolism; glyoxylate and dicarboxylate metabolism; glycine, serine, and threonine metabolism; and arginine biosynthesis.

**Conclusion:**

In summary, our results demonstrated that changes in the serum level of 25 metabolites may be useful in distinguishing CeD patients from healthy controls, which have the potential to be considered candidate biomarkers of CeD.

## 1. Introduction

Celiac disease (CeD), an autoimmune intestinal disorder that affects up to 1% of the world population, is caused by the ingestion of gluten (found in wheat, barley, and rye) in genetically predisposed individuals carrying HLA-DQ2 or HLA-DQ8 [1–3]. Gluten peptides trigger an immune reaction, which damages the small intestinal villi and causes nutrient malabsorption. Iron deficiency, osteoporosis, and bone disease, followed by mineral deficiencies, are known as celiac disease-associated disorders [4–7].

CeD diagnosis is mainly based on a combination of specific serological and histological evaluations. The small intestinal biopsy is considered the gold standard for adult CeD diagnosis. A certain level of expertise and skill is needed for the assessment of intestinal biopsies, and variability in sample quality and subjective interpretation can affect the diagnostic accuracy [8, 9]. Moreover, biopsy sampling is an invasive procedure and efforts are being made to find alternatives to this method [10]. In some cases, discrepancies between the clinical, histology, and serology findings make CeD diagnosis difficult. In particular, minor small bowel mucosal changes in latent CeD subjects usually lead to a misinterpretation [11]. The discovery of new diagnostic biomarkers can be a basis for the development of a point-of-care type of assays for monitoring celiac disease directly by the affected individual or by the healthcare professionals. Several approaches can be applied in identifying such novel celiac biomarkers, including microarray-based techniques, proteomics, and metabonomics. Metabolomics, genomics, transcriptomics, and proteomics can analyze living organisms and provide a better understanding of cellular biology. Each organism, organ, tissue, or cell has a characteristic metabolic profile that can be altered in response to pathophysiological stimuli or genetic modification [12]. Metabolomics can describe the biological changes by monitoring metabolites in body fluids and tissues and improve the understanding of the main mechanisms behind diseases. It can also help in the discovery of potential prognostic and diagnostic biomarkers of disease by describing biological changes between the target and control groups and give a cell physiology snapshot by metabolic profiling determination. In addition, metabolomics can provide a picture of cell function under specific conditions and physiological states [12–15]. Sometimes, tissue damage in diseases changes metabolic profiles in body fluids such as blood [12]. In 2015, Sharma et al. demonstrated that villus damage in the epithelial layer of the small intestine from CeD patients affects blood metabolic profile and proposed that measuring the altered levels of blood metabolites may provide a metabolic signature for intestinal damage [16].

Nuclear magnetic resonance (NMR) spectroscopy and mass spectrometry are useful analytical techniques to describe metabolic changes in response to disease, drugs, diet, toxins, and nutrient intake. The advantages of NMR spectroscopy include the need for a very small sample volume, simple preparation, high reproducibility, lower analysis time, and lower maintenance cost, providing complete information about a large number of metabolites [17]. Metabolomics based on NMR combined with multivariate data analysis has proven to be a very powerful method to determine changes in metabolite concentration in data consisting of a large number of samples.

As the current serologic tests for celiac disease can be accompanied by false-negative results (due to patients' IgA deficiency) or false-positive results (due to other autoimmune diseases and intercurrent infections) and since celiac disease is known as a pathology with a direct impact on metabolism [18–20], an NMR profile of serum metabolites may significantly improve the diagnosis process of the disease [20]. In particular, NMR-based metabonomic analysis of biological fluids can trace small changes of target metabolites, which doubled its importance [20]. Today, NMR is widely used to clarify the pathophysiology of different disorders (such as neurological disorders, cancer, gastrointestinal diseases, and cardiovascular disease) and identify diagnostic biomarkers for them [21–25]. Previous studies reported characteristic metabolic alterations of inflammatory bowel disease, fatty liver disease, *Helicobacter pylori* infection, etc. [20, 26, 27].

Bertini et al. introduced some metabolomics biomarkers in the serum and urine of CeD patients which were significantly different between healthy controls and patients. In addition, in this study, the serum and urine of CeD patients were examined after 12 months of GFD and no significant variations in levels of other resonances were found between patients and healthy subjects [20].

In this study, we compared serum metabolites of untreated CeD patients and healthy controls to introduce novel reliable diagnostic biomarkers for CeD screening.

## 2. Materials and Methods

### 2.1. Study Population and Sample Collection

Forty-two active adult CeD patients, who did not start a gluten-free diet (22 females and 20 males with a mean age of 33 ± 10 years (mean ± SD)), were recruited from the Research Center for Gastroenterology and Liver Diseases at the Taleghani Hospital from August 2019 to February 2020. The CeD diagnosis was based on positive serology (anti-endomysial (EMA) and anti-transglutaminase-2 (TG2) antibodies) confirmed by villous abnormalities subclassified into modified Marsh grade ≥ 2 [28]. Patients with positive tTGA and EMA serology tests but with Marsh 0/1 lesions were excluded from the analysis. Pregnant and lactating women, patients with any other autoimmune/gastrointestinal diseases, and patients who had a history of nonsteroidal anti-inflammatory drug intake were excluded too. Twenty-two gender- and age-matched healthy controls (10 females and 12 males with a mean age of 35 ± 12 years (mean ± SD)) were also recruited as the control group. Both CeD subjects and healthy controls had no significant past medical history such as hypertension or diabetes mellitus (Table 1).

This study was approved by the ethical committee of Shahid Beheshti University of Medical Sciences, Tehran, Iran (IR.SBMU.RETECH.REC.1399.1146). Participants were informed about the content of the study, and written consent was signed by all of them.

Blood samples (5 mL) were collected in the morning after overnight fasting into plastic serum tubes. The tubes were placed vertically at room temperature (22°C) for 20 minutes and centrifuged at 10,000 rpm using an Eppendorf centrifuge at room temperature for 10 minutes. The serum supernatant was removed into a fresh polypropylene tube, immediately frozen, and stored at -80°C until NMR analysis.

### 2.2. ^1^H NMR Spectroscopy

Acquiring NMR is similar to our previous work with a new analysis [29–31]. Hydrogen-1 NMR or ^1^H NMR spectroscopy analysis of serum samples was performed using a Bruker Avance 400 spectrometer, operating at 400 MHz ^1^H resonance frequency. A 5 mm high-quality NMR tube (Sigma-Aldrich, RSA) was used.

After inserting 10% D_2_O (deuterium oxide, 99.9%D, Aldrich Chemicals Company) into the serum sample of each individual, the ^1^H NMR spectra were acquired immediately and referenced to the chemical shift of lactate at *δ* = 1.33. The D_2_O provided a field-frequency lock solvent for the NMR spectrometer. Typically, ^1^H NMR spectra were measured with the following parameters: spectral width: 8389.26 Hz; time-domain points: 32 K; number of scans: 154; acquisition time: 2 s; spectrum size: 32 K; and line broadening: 0.3 Hz. In order to enhance visualization of the low-molecular weight metabolites and to assuage protein and lipoprotein's broad signals, the Carr-Purcell-Meiboom-Gill (CPMG) pulse sequence was applied for serum sample analysis [32–34].

### 2.3. Data Preprocessing

Phase and baseline distortions were manually corrected for all ^1^H NMR spectra within XWINNMR (version 3.5, Bruker Spectrospin Ltd.). ^1^H NMR spectral processing (baseline correction, normalization, and alignment) was performed using ProMetab software (version prometab_v3_3) [35] in MATLAB (version 6.5.1, MathWorks, Cambridge, UK). In order to remove the effects of the residual water peak in the region, *δ*_1H_ = 4.5-5.5 ppm was set to zero in all NMR spectra. This software integrates the bins across the spectral regions of 0.02 ppm width within the range of 0.2 and 10.0 ppm. Then, baseline correction and alignment were done by ProMetab software in MATLAB. To decrease any significant concentration differences between samples, data were mean-centered and Pareto-scaled after importing data into SIMCA.

## 3. Statistical Analysis

SIMCA software version 14.0 (Umetrics, Umeå, Sweden) and SPSS 16.0 (SPSS, Inc., Chicago, IL) were used for analyzing metabolomics results. SIMCA is used widely as a commercial tool in metabolomics data analysis. Principal component analysis (PCA) was applied as an unsupervised statistical method to find outliers, patterns, and trends within the dataset and visualize intrinsic clusters [36]. Also, Orthogonal Projections to Latent Structures Discriminant Analysis (OPLS-DA) was performed as a supervised statistical method on NMR data using SIMCA to identify metabolite fingerprint differences and construct predictive models.

*R* ^2^ *X*, *R*^2^*Y*, and *Q*^2^, three goodness parameters for the OPLS-DA model, were calculated using the default leave-one-out (LOO) procedure to describe the quality of the OPLS-DA model [37]. To evaluate the OPLS-DA prediction performance, the receiver operating characteristic (ROC) curve was used and the area under the ROC (AUC) value was calculated using SPSS 16.0 (SPSS, Inc., Chicago, IL). Specificity and sensitivity were determined according to the prediction of the sample class using the 7-fold cross-validation [38].

## 4. Metabolite Identification

Identification of metabolites was done manually based on signal multiplicity and assignments, which were published in the literature [39, 40], and online databases such as the Biological Magnetic Resonance Data Bank (BMRB) (http://www.bmrb.wisc.edu/metabolomics/) [41] and the Human Metabolome Database (HMDB) (http://hmdb.ca/) [42].

## 5. Metabolic Pathway Analysis

By MetaboAnalyst 4.0 (accessible at http://www.metaboanalyst.ca/), a simple and freely available tool that combines pathway enrichment analysis and topology analysis, metabolic pathway analysis was performed. The online software MetaboAnalyst with 6292 metabolite sets, 15 model organisms, and three types of biofluids (cerebrospinal fluid, blood, and urine) was used widely in metabolomics studies [43, 44].

The metabolic pathways that are used by MetaboAnalyst are the basis of the Kyoto Encyclopedia of Genes and Genomes (KEGG) database. Identified metabolites by NMR, which showed significant differences between celiac patients' serum samples and healthy controls, were entered into MetaboAnalyst. The Homosapiens library, hypergeometric test default, and relative betweenness centrality algorithms were chosen as the options for the enrichment analysis and pathway topology analysis. In each pathway, the numbers of involved metabolites (hits) were reported. The most important pathways, with*p*values and false discovery rates (FDR) less than 0.05, were considered significant [45].

## 6. Results

### 6.1. Comparison of Altered Serum Metabolic Profiles between CeD Patients and Healthy Controls

**Figure 1:**
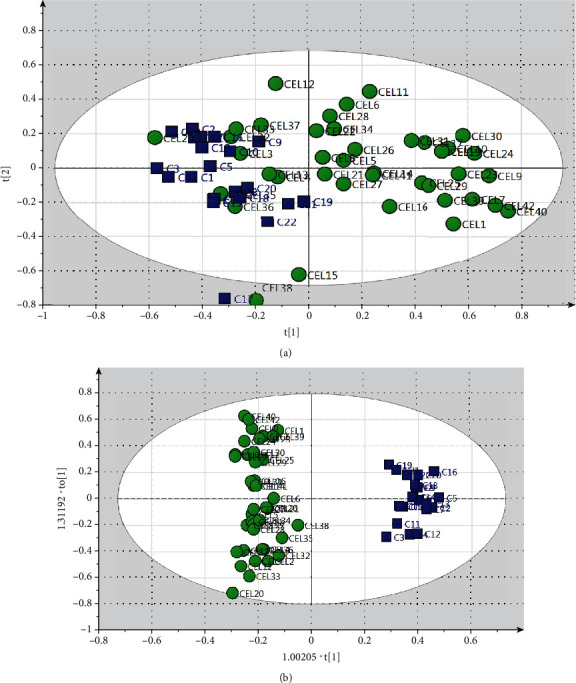
Multivariate statistical analysis from NMR-based metabolic profiling. (a) PCA score plot with all variable unit variance scaled. (b) OPLS-DA score plot of the CeD group versus the healthy control group. Circle, CeD; square, healthy controls.

After NMR spectral preprocessing, the resulting binned data including 64 samples and 408 variables were analyzed by unsupervised PCA to find patterns, trends, and outliers. Two samples from CeD patients were located far away from the 99% Hotelling's *T*^2^ confidence limit and were considered outliers. After excluding two outliers, PCA was carried out again. PCA score plots showed that the CeD group is not separated clearly from the healthy control group (*R*^2^*X*: 0.817; *Q*^2^: 0.58) (Figure 1(a)). Then, OPLS-DA was performed to detect alterations between two groups and identify the different metabolic patterns and the potential biomarkers. The OPLS-DA score plots (*R*^2^*X*: 0.603; *R*^2^*Y*: 0.967; *Q*^2^: 0.93; and *p* value: 7.51*E*-28) showed that the CeD group was distinct from healthy controls.

To further validate the diagnostic performance, the ROC curve was used and the calculated AUC value was 1 in this model (Supplementary figure (available here). These results indicated that the OPLS-DA model had a high predictive power between the CeD group and the healthy control group, showing that NMR-based fingerprinting could be used to differentiate CeD subjects from healthy controls.

Metabolites responsible for separating CeD samples from healthy controls in the OPLS-DA model are shown in Table 2. Data analysis based on their chemical shifts and signal multiplicity according to online databases (http://hmdb.ca) and the literature showed changes in 25 different metabolites which were related to the following: amino acids (glutamine (Gln), isoleucine (Ilu), lysine (Lys), valine (Val), proline (Pro), serine (Ser), and glutamic acid (Glu)); bile acids (chenodeoxycholic acid (CDCA), taurocholic acid (TCA), cholic acid (CA), glycocholic acid (GCA), and lithocholic acid (LC)); fatty acids (elaidic acid (EA), linoleic acid (LIN), stearic acid (SA), and propionic acid (PA)); triglycerides (tg); glucose (Glc); cholesterol; 3-hydroxyisobutyric acid (3-HIB); isobutyrate; betaine (Bet); taurine; choline (Cho); and acetylcholine (Ach) between CeD and healthy controls. NMR spectroscopy of serum samples showed higher levels of glucose, bile acids, betaine (Bet), taurine, 3-HIB, isobutyrate, and Ach and reduced levels of Gln, Ilu, Lys, Val, Pro, Ser, Glu, fatty acids (EA, LIN, SA, and PA), cholesterol, and triglycerides in specimens of CeD patients than healthy controls.

### 6.2. Metabolic Pathway Analysis of Altered Profiles

**Figure 2:**
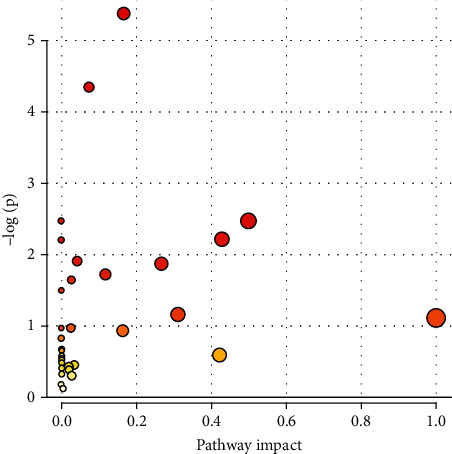
Pathway analysis overview showing altered metabolic pathways in serum from CeD subjects using MetaboAnalyst 4.0. (1) Aminoacyl-tRNA biosynthesis. (2) Primary bile acid biosynthesis. (3) Nitrogen metabolism. (4) Glutamine and glutamate metabolism. (5) Valine, leucine, and isoleucine biosynthesis and degradation. (6) Taurine and hypotaurine metabolism. (7) Glyoxylate and dicarboxylate metabolism. (8) Glycine, serine, and threonine metabolism. (9) Arginine biosynthesis.

**Figure 3:**
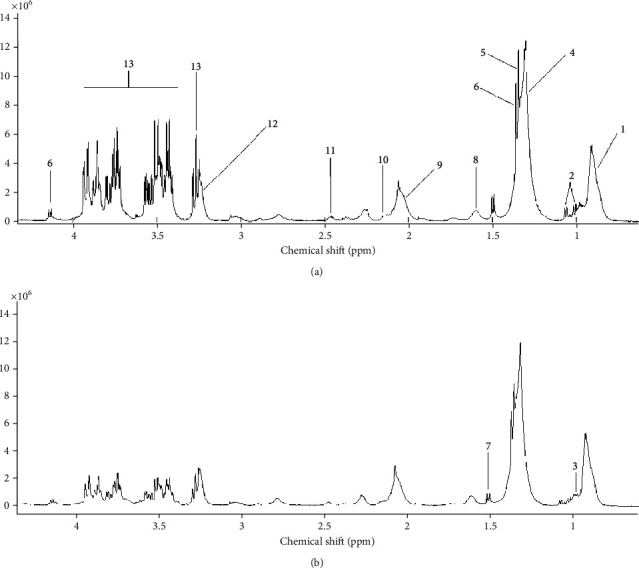
Representative 400 MHz one-dimensional CPMG ^1^H NMR spectrum of celiac disease (a) and healthy control (b) subjects. (1) Lipid: LDL CH_3_-(CH_2_)*_n_*. (2) Valine. (3) Leucine. (4) Lipid: VLDL (CH_2_)*_n_*-CO. (5) Threonine. (6) Lactate. (7) Alanine. (8) Lipid: VLDL CH_2_-CH_2_-CO. (9) Lipid: CH_2_-CH=CH. (10) Glutamate+glutamine. (11) Glutamine. (12) Choline. (13) *α-*Glucose and *β*-glucose.

Based on the identified metabolites in serum, metabolic pathways were investigated by applying the MetaboAnalyst 3.0 server. Nine metabolic pathways including aminoacyl-tRNA biosynthesis; primary bile acid biosynthesis; nitrogen metabolism; glutamine and glutamate metabolism; valine, leucine, and isoleucine biosynthesis and degradation; taurine and hypotaurine metabolism; glyoxylate and dicarboxylate metabolism; glycine, serine, and threonine metabolism; and arginine biosynthesis were altered in CeD serum samples (Figures 2 and 3).

Statistics related to pathways with major changes based on *p* value and FDR indicated that only two pathways (aminoacyl-tRNA biosynthesis and primary bile acid biosynthesis) showed *p* value < 0.05 and FDR < 0.05, whereas seven pathways showed only *p* value < 0.05 (Table 3).

## 7. Discussion

Blood biochemical composition is known as the main basis of clinical biochemistry to describe pathological conditions. Potential biomarkers associated with different diseases can be described by metabolite analysis of biological samples. Moreover, using the obtained profile may be helpful in achieving a better understanding of the disease pathogenesis and mechanism from a holistic point of view.

As the metabolome shows alteration of both the genome and the proteome, an NMR-based metabolic profile, when paired with a transient statistical analysis, provides a comprehensive metabolic picture of such a multifactorial pathology. CeD metabolomics may identify new molecular mechanisms, which can clarify CeD-related symptoms as currently there is no explanation for them. In this study, we analyzed CeD patients' and healthy controls' serum metabolite levels and found that there is a distinct pattern between*t*values and a metabolic signature for CeD in serum samples from celiac patients.

Our results showed changes in the level of 25 metabolites, which can be useful in distinguishing CeD patients from the healthy control group (Table 2). These altered metabolites are related to lipid, carbohydrate, and amino acid metabolism.

Higher levels of glucose in celiac samples can be related to the upregulation of glucose intake at the microvillus membrane surface due to their altered lipid-to-protein ratio and impairment of one or more steps in the glycolysis process [20].

When glycolysis is disturbed and reduced, fatty acid *β*-oxidation, the second major metabolic pathway responsible for energy supply, is usually overstimulated to produce energy. Malabsorption and the increase of fatty acid *β*-oxidation explain lower fatty acid levels in CeD serum [46].

In gluten metabolism conditions, amino acids can also be used as energy sources and affect cellular metabolism and immune system signaling [47, 48]. In glycolysis impairment, the amino acid carbon backbone can convert into citric acid cycle intermediates or their precursors to provide energy. Therefore, the decrease in serum concentration of amino acids of CeD cases can be due to the decreased amino acid absorption as a result of villous atrophy and their participation in energy production [49, 50].

We demonstrated that the isobutyrate and 3-HIB levels were increased and the FA level was decreased in CeD serum samples when compared to healthy controls. 3-HIB, an intermediate of valine catabolism, is secreted from muscle cells, enhances muscle lipid accumulation, regulates endothelial fatty acid (FA) transport, and connects the regulation of FA flux to catabolism of the branched-chain amino acids (BCAAs; valine, leucine, and isoleucine) [51]. Decreased glycolysis and increased fatty acid oxidation caused higher levels of BCAA. High activity of BCAA aminotransferase increased BCAA catabolism in muscles [52]. Increased levels of 3-HIB in CeD subjects can be related to the increased catabolic flux of BCAAs or microbial activity. Increased BCAA catabolic flux causes 3-HIB secretion from muscle and imports excessive transendothelial FA into the muscle. Elevated 3-HIB levels indicate secretion of BCAA catabolic flux and regulate metabolic flexibility in muscles and the heart. It has been shown that 3-HIB can be used as a risk indicator of insulin resistance (IR) and the future development of type 2 diabetes (T2D) [51, 53].

Isobutyrate and 3-HIB are produced from glucose and amino acids (valine) in gut microbiota. The gut microbiota, formed by a large number of microorganisms, produces some compounds under the influence of environmental stimuli and affects the host metabolome and its health. According to the studies, the gut microbiota composition, which can be influenced by genetic factors including HLA molecules, may have a role in the development of several immune-based disorders. A limited number of studies have reported a link between alterations in the gut microbiota and the onset of intestinal diseases such as inflammatory bowel disease and celiac disease. For instance, Bodkhe et al. reported significant decreases in *Lactobacillus sakei* and total *Lactobacillus* populations in GFD-treated celiac patients compared to untreated and healthy subjects [54]. Serena et al. in their study observed the change in blood microbiome composition and taxonomic diversity in the samples of adult CeD subjects compared with healthy controls [55]. Moreover, Leonard and coworkers using the Celiac Disease Genomic, Environmental, Microbiome, and Metabolomic (CDGEMM) study, which is about understanding the role of the gut microbiome as an additional factor in the susceptibility to autoimmune diseases, revealed that several microbial species, functional pathways, and metabolites might be specific to CeD [56, 57]. In this regard, Olshan et al. in a recent study reported significant differences at both the strain level and the species level for bacteria and viruses and in functional pathways in breast milk composition of subjects with CeD on a gluten-free diet than healthy controls [58]. Rheumatoid arthritis and celiac disease are two diseases with similarities such as HLA mutations that show similar microbial dysbiosis, which can lead to worsening both diseases' severity [59].

Gut microbiota productions can be absorbed by the colonic epithelium; they enter the bloodstream and play an important role in regulating the metabolism of glucose, fatty acids, and cholesterol. Changes in serum metabolite levels in CeD have been identified and suggest significant changes in the gut microbiota of CeD subjects [60–65].

The reduction of cholesterol concentration in CeD patients than healthy controls can be related to intestinal malabsorption, decreased cholesterol genesis, increased bile acid biosynthesis, and elimination of high-cholesterol feces. Failure to increase cholesterol levels in CeD patients under treatment indicates that intestinal malabsorption is less involved in this process [66]. We indicated that bile acids such as chenodeoxycholic acid, taurocholic acid, cholic acid, glycocholic acid, and lithocholic acid have a higher concentration in CeD serum samples in comparison to healthy controls. These compounds and taurine are synthesized from cholesterol during the primary bile acid biosynthesis mechanism [66–69].

The size of the bile acid pool is affected by the microbial metabolism of bile acids in the intestines. Bile acids regulate the gut microbiome at the highest toxemic levels. The host and microbiome regulate the size of the bile acid pool. A large pool of a conjugate of hydrophilic bile acids is produced by the host. The members of the microbiome use bile acids and their compounds. Bacterial overgrowth, inflammation, antibiotic therapy, diet (such as gluten-free diet), and disease states affect the microbiome-bile acid pool balance [70].

We demonstrated that Bet and choline have a high concentration in the CeD group compared to healthy controls. Choline plays a critical role in lipid metabolism and methylation [71]. It is an essential component of the lipids present in the plasmatic membrane and structural lipoproteins and a precursor of acetylcholine. Bet is synthesized from choline oxidation and glycine during glycine, serine, and threonine metabolism. Choline and Bet are important sources of one-carbon units and are involved in the pathogenesis of various disorders such as chronic diseases and neurological developmental disorders [72]. High choline concentrations in plasma are associated with cardiovascular risk factor profiles. Moreover, altered choline metabolite levels may act as biomarkers for changes in membrane metabolism in CeD patients. Choline and Bet have opposite relationships with the major components of metabolic syndrome and have a key role in disease prevention and risk assessment. The presented results indicate their involvement in the pathogenesis of various chronic diseases [72, 73].

Higher levels of ACh in CeD may be related to increased acetylcholine biosynthesis from acetyl-CoA and inhibition or inactivation of acetylcholinesterase (AChE), the enzyme responsible for the ACh degradation in cholinergic neurons [74]. ACh, a neurotransmitter, is synthesized from choline and acetyl-CoA. High levels of acetyl-CoA are obtained from the *β*-oxidation cycle. Excessive accumulation of ACh at the synapses and neuromuscular junctions causes symptoms of both nicotinic toxicity and muscarinic toxicity. Fatigue and muscle weakness in CeD may be an immune-mediated neurological disorder, which is caused by an increase in Ach concentration [75, 76].

These biomarkers still need confirmation through additional techniques, such as LC/MS and 2D NMR. Moreover, in the present study, metabolic pattern differences between CeD and other gastrointestinal diseases have not been studied. These were our study limitations. Further studies with other techniques and in different societies are needed to confirm/reject the result of our study.

## 8. Conclusion

Analysis of CeD patients' and healthy controls' serum metabolite levels showed that changes in the serum level of 25 metabolites can be useful in distinguishing CeD patients from the healthy control group and may be considered candidate biomarkers of CeD, which needs to be confirmed by the results of subsequent studies. Our results may further enhance the understanding of impaired metabolic pathways in CeD.

## Figures and Tables

**Table 1:** Baseline demographics of the study participants.

Demographic factors	Study groups	
	Celiac disease patients (*n* = 42)	Healthy controls (*n* = 22)
	Mean ± SE	Mean ± SE
Age (years)	33 ± 10	35 ± 12
Males	20 (47.6%)	12 (54.5%)
Females	22 (52.4%)	10 (45.5%)
Baseline height (cm)	159 ± 33	162 ± 22
Baseline weight (kg)	56 ± 43	61 ± 21
Marsh classification		
Marcsh 2	9 (21.5%)	0
Marsh 3	33 (78.5%)	0

**Table 2:** Differential serum metabolites between CeD samples and healthy controls using NMR.

No.	Metabolite	*δ*_1H_ (ppm)^a^	Fold change^b^	Direction of variation^c^	Biochemistry pathway
1	Glycocholic acid	3.47	1.49	↑	Primary bile acid biosynthesis
2	Chenodeoxycholic acid	1.99, 1.97, 2.01	1.59	↑	Primary bile acid biosynthesis
3	Glucose	3.35, 5.23, 3.83, 3.39	1.61	↑	Glycolysis
4	Betaine	3.89	1.67	↑	Choline oxidation Glycine, serine, and threonine metabolism
5	Taurine	3.41	1.70	↑	Primary bile acid biosynthesis
6	Taurocholic acid	0.87, 0.81, 0.83, 0.85	1.71	↑	Primary bile acid biosynthesis
7	Choline	3.19	1.74	↑	Lipid metabolism
8	Cholic acid	1.27	1.89	↑	Primary bile acid biosynthesis
9	Acetylcholine	3.21	2.15	↑	Acetylcholine biosynthesis
10	Lithocholic acid	1.25	2.33	↑	Primary bile acid biosynthesis
11	3-Hydroxyisobutyric acid	1.11	3.56	↑	BCAA catabolism Gut microbiota
12	Isobutyrate	1.13	9.1	↑	Gut microbiota
13	Glutamine	2.47	1.55	↓	Amino acid metabolism
14	Elaidic acid	1.59	1.50	↓	Fatty acid metabolism
15	Linoleic acid	1.35	1.65	↓	Fatty acid metabolism
16	Isoleucine	0.93	2.28	↓	Amino acid metabolism
17	Triglycerides	4.15	1.67	↓	Lipid metabolism
18	Lysine	3.73	1.48	↓	Amino acid metabolism
19	Stearic acid	1.37	2.12	↓	Fatty acid metabolism
20	Cholesterol	3.51, 0.91	1.42	↓	Steroid biosynthesis
21	Valine	1.01	1.57	↓	Amino acid metabolism
22	Proline	2.09	1.41	↓	Amino acid metabolism
23	Propionic acid	1.07	2.05	↓	Fatty acid metabolism
24	Serine	3.93	1.51	↓	Amino acid metabolism
25	Glutamic acid	2.07	1.94	↓	Amino acid metabolism

Abbreviations: BCAA: branched-chain amino acid; NMR: nuclear magnetic resonance. ^a^Chemical shift scale of the NMR signal used for the quantification of metabolites. ^b^Fold change for each chemical shift was calculated based on the median values. ^c^Increased or decreased metabolites in the CeD group compared with the healthy control group.

**Table 3:** Significant pathway based on *p* values/FDR.

	Pathway name	Total^a^	Hits^b^	*p*	−log(*p*)	FDR	Impact^c^
1	Aminoacyl-tRNA biosynthesis	48	7	4.058*E*-6	5.3917	3.4087*E*-4	0.16667
2	Primary bile acid biosynthesis	46	6	4.4108*E*-5	4.3555	0.0018525	0.07433
3	Nitrogen metabolism	6	2	0.0033199	2.4789	0.069718	0.0
4	D-Glutamine and D-glutamate metabolism	6	2	0.0033199	2.4789	0.069718	0.5
5	Valine, leucine, and isoleucine biosynthesis	8	2	0.0060807	2.216	0.08513	0.0
6	Taurine and hypotaurine metabolism	8	2	0.0060807	2.216	0.08513	0.42857
7	Glyoxylate and dicarboxylate metabolism	32	3	0.012055	1.9188	0.13782	0.04233
8	Glycine, serine, and threonine metabolism	33	3	0.013126	1.8819	0.13782	0.26741
9	Arginine biosynthesis	14	2	0.018672	1.7288	0.17427	0.11675

Abbreviations: FDR: false discovery rate. ^a^The total number of metabolites in each pathway. ^b^The number of identified metabolites in each pathway. ^c^The pathway impact is based on scores from topology analysis.

## Data Availability

The data used to support the findings of this study are available from the corresponding author upon request.

## Supplementary Materials

Supplementary Materials Supplementary Figure 1: ROC curve analysis for the predictive power of serum biomarkers for distinguishing CeD from healthy controls using the OPLS-DA model. Serum metabolite biomarkers by OPLS-DA yielded an AUC value of 1.
