# Supplementary figures and images for: Screening of Altered Metabolites and Metabolic Pathways in Celiac Disease Using NMR Spectroscopy
Source: Biomed Res Int. 2021 Nov 15;2021:1798783. doi: 10.1155/2021/1798783 (PMC8608527; doi:10.1155/2021/1798783)

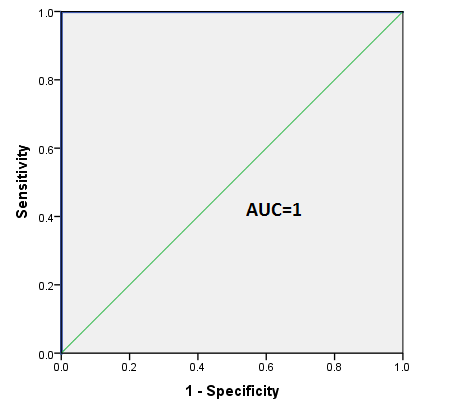

Supplement: Supplementary Materials — Supplementary Figure 1: ROC curve analysis for the predictive power of serum biomarkers for distinguishing CeD from healthy controls using the OPLS-DA model. Serum metabolite biomarkers by OPLS-DA yielded an AUC value of 1. [file 1798783.f1.tif]
